# CXCR6-positive circulating mucosal-associated invariant T cells can identify patients with non-small cell lung cancer responding to anti-PD-1 immunotherapy

**DOI:** 10.1186/s13046-024-03046-3

**Published:** 2024-05-03

**Authors:** Jingjing Qu, Binggen Wu, Lijun Chen, Zuoshi Wen, Liangjie Fang, Jing Zheng, Qian Shen, Jianfu Heng, Jianya Zhou, Jianying Zhou

**Affiliations:** 1 Department of Respiratory Disease, Thoracic Disease Center, The First Affiliated Hospital, Zhejiang University School of Medicine, Hangzhou, Zhejiang 310003 P. R. China; 2 The Clinical Research Center for Respiratory Diseases of Zhejiang Province, Hangzhou, Zhejiang 310003 P. R. China; 3 State Key Laboratory for Diagnosis and Treatment of Infectious Diseases, National Clinical Research Center for Infectious Diseases, The First Affiliated Hospital, Zhejiang University School of Medicine, Hangzhou, Zhejiang 310003 P.R. China; 4 Department of Cardiology, The First Affiliated Hospital, The First Affiliated Hospital, Zhejiang University School of Medicine, Hangzhou, Zhejiang 310003 P. R. China; 5 Department of Clinical Pharmaceutical Research Institution, Hunan Cancer Hospital/the Affiliated Cancer Hospital of Xiangya School of Medicine, Central South University, Changsha, Hunan 410013 P. R. China

**Keywords:** Circulating mucosal-associated invariant T cells, CXCR6, Non-small cell lung cancer, Single-cell RNA-sequencing, Immunotherapy

## Abstract

**Background:**

Mucosal-associated invariant T (MAIT) cells have been reported to regulate tumor immunity. However, the immune characteristics of MAIT cells in non-small cell lung cancer (NSCLC) and their correlation with the treatment efficacy of immune checkpoint inhibitors (ICIs) remain unclear.

**Patients and methods:**

In this study, we performed single-cell RNA sequencing (scRNA-seq), flow cytometry, and multiplex immunofluorescence assays to determine the proportion and characteristics of CD8+MAIT cells in patients with metastatic NSCLC who did and did not respond to anti-PD-1 therapy. Survival analyses were employed to determine the effects of MAIT proportion and C-X-C chemokine receptor 6 (CXCR6) expression on the prognosis of patients with advanced NSCLC.

**Results:**

The proportion of activated and proliferating CD8+MAIT cells were significantly higher in responders-derived peripheral blood mononuclear cells (PBMCs) and lung tissues before anti-PD-1 therapy, with enhanced expression of cytotoxicity-related genes including CCL4, KLRG1, PRF1, NCR3, NKG7, GZMB, and KLRK1. The responders’ peripheral and tumor-infiltrating CD8+MAIT cells showed an upregulated CXCR6 expression. Similarly, CXCR6+CD8+MAIT cells from responders showed higher expression of cytotoxicity-related genes, such as CST7, GNLY, KLRG1, NKG7, and PRF1. Patients with ≥15.1% CD8+MAIT cells to CD8+T cells ratio and ≥35.9% CXCR6+CD8+MAIT cells to CD8+MAIT cells ratio in peripheral blood showed better progression-free survival (PFS) after immunotherapy. The role of CD8+MAIT cells in lung cancer immunotherapy was potentially mediated by classical/non-classical monocytes through the CXCL16-CXCR6 axis.

**Conclusion:**

CD8+MAIT cells are a potential predictive biomarker for patients with NSCLC responding to anti-PD-1 therapy. The correlation between CD8+MAIT cells and immunotherapy sensitivity may be ascribed to high CXCR6 expression.

**Supplementary Information:**

The online version contains supplementary material available at 10.1186/s13046-024-03046-3.

## Introduction

Lung cancer remains the leading cause of cancer-related deaths worldwide. It is the second most diagnosed cancer type according to global cancer statistics in 2020 [1]. Non-small cell lung cancer (NSCLC) is the most common type of lung cancer, accounting for approximately 85% of all cases. NSCLC is further categorized into three subtypes based on specific histological characteristics, including adenocarcinoma, squamous cell carcinoma, and large-cell carcinoma [2, 3].

Owing to the high incidence and mortality of lung cancer, more and more studies have attempted to explore new therapeutic strategies, including immunotherapy. Immunotherapy, which mainly includes immune-checkpoint inhibitors (ICIs), cellular immunotherapy for cancer, cancer-specific vaccines, and other immune system modulators, is a type of cancer treatment that helps the immune system recognize and eliminate malignant cells [4, 5]. Programmed cell death 1 (PD-1)/programmed cell death ligand 1 (PD-L1) checkpoint inhibitors are commonly used ICIs for patients with NSCLC. However, not all patients with NSCLC are suitable candidates for PD-1/PD-L1 blockade therapy. PD-L1 expression in tumors is a crucial factor determining the efficacy of PD-1/PD-L1 checkpoint inhibitors in NSCLC therapy [6, 7]. However, some patients with low PD-L1 expression respond well to immunotherapy, whereas others with high PD-L1 expression do not. Therefore, it is important to identify definitive predictive markers to determine whether PD-1/PD-L1 checkpoint inhibitors are appropriate treatment options.

The efficacy of ICIs is closely related to tumor microenvironment (TME)[8]. The TME refers to a complex ecosystem comprising cells, extracellular matrix, and signaling molecules surrounding the tumor. It is closely associated with tumorigenesis and tumor development. The TME mainly includes tumor cells, stromal cells, tumor-associated fibroblasts, immune cells, extracellular matrix, and tumor blood vessels [9]. The complex interactions between these components in the TME are crucial for the growth of malignant tumors. An in-depth study of the composition and function of the TME helps understand the mechanism of tumor occurrence, thereby assisting in developing new treatment strategies and improving patient prognosis [10, 11]. The development of single-cell RNA sequencing (scRNA-seq) technology has provided researchers with an important tool to explore the TME better. scRNA-seq has been widely applied in various aspects of cancer research [12, 13]. In this study, we conducted scRNA-seq analysis to explore the genetic characteristics of immunotherapy-responsive and non-responsive patients with NSCLC.

Mucosal-associated invariant T (MAIT) cells are a type of unconventional T cell. It is a relatively conserved subtype, expressing an invariant T cell receptor (TCR) α chain Vα7.2-Jα33. It is enriched in mucosal tissues and non-mucosal tissues, such as the blood, liver, and lung [14]. The semi-invariant TCR of MAIT cells can specifically combine with the riboflavin derivatives presented by MR1, thus mediating the activation of MAIT cells[15]. Once activated, MAIT cells can produce various cytokines, such as INF-γ and TNF, leading to cytotoxic effector functions[16]. MAIT cells are crucial in the defense against microbial invasion through an innate-like immune response. Besides, growing studies have demonstrated the correlation between MAIT cells and the tumor immune microenvironment. Previous researches have shown that the number of MAIT cells were reduced in the peripheral blood of patients with solid tumors, including lung cancer, and tumor-infiltrating MAIT cells may exhibit impaired cytotoxic effector functions [17]. Nevertheless, the role of MAIT cells in the tumor immune microenvironment is still poorly understood, and it is unclear whether MAIT cells can serve as new biomarkers for immunotherapy efficacy in patients with NSCLC.

C-X-C chemokine receptor 6 (CXCR6) is a chemokine receptor. CXCR6 is located on chromosome 3p21.31 and has four splice isoforms. The translational product of CXCR6 is a G-protein-coupled receptor with seven transmembrane domains, belonging to the CXC chemokine receptor family and is mainly expressed in certain subsets of T cells. CXCL16 is a unique ligand of CXCR6 in both membrane-bound and soluble forms. CXCL16 is predominantly expressed in epithelial cells and antigen-presenting cells such as dendritic cells [18]. CXCR6 is necessary for the recruitment and long-term residence of resident memory T cells (T_RM_) [19–21]. The deficiency of CXCR6 or CXCR6-CXCL16 interaction leads to tumor proliferation [22, 23] and lower anti-tumor efficacy in tumor vaccination therapy, which is mainly based on T_RM_ infiltration [24]. CXCR6 + CD8 + T cells are more immunocompetent and can be recruited to the TME to exert cytotoxic effects, leading to tumor cell destruction[23, 25]. In lung cancer, CXCR6 affects the recruitment of antitumor CD8 + T cells to the TME[24], and low expression of CXCR6 significantly correlates with poor prognosis in patients with lung cancer[25]. However, the expression profiles and characteristics of CXCR6 in MAIT cells remain unclear. The correlation between CXCR6-CXCL16 and anti-PD-1/PD-L1 therapy is still unknown.

In this study, we performed scRNA-seq analysis of peripheral blood samples from anti-PD-1 therapy responders or non-responders among patients with NSCLC. CXCR6 was significantly upregulated in responders-derived CD8+MAIT cells, which were specifically enriched in patients with NSCLC sensitive to anti-PD-1/PD-L1 therapy. We found that CXCR6+CD8+MAIT cells exhibited activated cytotoxic effector functions. Our results, for the first time, reveal that CXCR6 expression in CD8+MAIT cells may be associated with the sensitivity of patients with NSCLC to immunotherapy and shed light on new predictive biomarkers for anti-PD-1/PD-L1 therapy.

## Methods

### Patient samples

This study was approved by the Clinical Research Ethics Committee of the First Affiliated Hospital, School of Medicine, Zhejiang University (No. 2023 − 0260). The whole blood samples for the scRNA-seq analysis of advanced NSCLC patients treated with pembrolizumab plus chemotherapy as first-line treatment at baseline (before immunotherapy) were collected. The responses of NSCLC patients to immunotherapy were assessed in accordance with The Response Evaluation Criteria in Solid Tumors (RECIST) clinical practice guidelines[26]. Patient responses to immunotherapy were evaluated at a 6-week timepoint (before the third cycle of treatment). The patients were categorized into two groups: responders (R) and non-responders (NR). Responders were identified as individuals exhibiting a RECIST response, including complete response (CR), partial response (PR), and stable disease (SD) lasting more than 6 months without progression. Non-responders, on the other hand, were composed of progressive disease (PD) and SD lasting less than 6 months before disease progression [27].

### Single-cell preparation

The whole blood sample was mixed by gently inverting the tube; 2 ml of the blood sample was transferred to a centrifuge tube, and PBS (HyClone) was added to make up the volume to 4 ml. After mixing fully, transferred the diluted blood sample to the upper layer of a new centrifuge tube with 3 ml Ficoll paque-Plus carefully. The tube was centrifuged at 1000 × g for 40 min at 18-20 ℃, and then the upper layer (plasma and PBS) was removed. Transferred the second layer (peripheral blood mononuclear cells, PBMCs) to a new centrifuge tube and PBS was added to make up the volume to 10 ml. Centrifuged at 400 × g for 10 min and removed supernatant. 1 ml PBS and 2 ml red blood cell lysis buffer (Singleron) was added to the tube, and the mixture was incubated for 5–8 min at room temperature to remove red blood cells. The tube was centrifuged at 300 × g for 5 min and the supernatant was removed. The samples were resuspended in PBS and centrifuged at 300 × g for 5 min. After resuspension in PBS, cell viability and density were analyzed microscopically after trypan blue staining (Sigma-Aldrich).

### scRNA-seq analysis

The prepared single-cell suspension was stored in PBS at a final cell concentration of 2 × 10^5^ cells/mL and then loaded onto a microwell chip of the Singleron Matrix Single Cell Processing System. Subsequently, barcoded Beads were retrieved from the microwell chip for mRNA capture. After barcoding, reverse transcription of the captured mRNA was performed to generate cDNA, followed by PCR amplification. The amplified cDNA was fragmented and ligated using sequencing adapters. The Single-Cell RNA Library Kit (Singleron, China) was used following the manufacturer’s instructions to construct scRNA-seq libraries. Finally, we diluted the individual libraries to a final concentration of 4 nM and performed sequencing on the Illumina NovaSeq 6000 sequencing platform with 150 bp paired-end reads.

### Flow cytometry

The whole blood samples were collected from patients with NSCLC who were responsive or non-responsive to immunotherapy at baseline. PBMCs were obtained using Ficoll -based density gradient centrifugation. For flow cytometry analysis, PBMCs were pretreated with a human IgG blocker (Cat #422302; BioLegend) for 20 min. Then the cells were washed thrice with FACS buffer (including 1 × PBS + 0.5% BSA), and centrifuged at 800 × *g* and 4 ℃ for 5 min. Specific fluorescently-labeled extracellular antibodies diluted in FACS buffer were added and incubated for 30 min in the dark on ice for staining. After washing and resuspension in FACS buffer, samples were analyzed using a BriCyte E6 flow cytometer (Mindray). Antibodies used were as follows: APC/Cyanine7 anti-human CD3 antibody (Biolenged Cat# 300425), PerCP/Cyanine5.5 anti-human CD8a antibody (Biolenged Cat# 301031), PE/Cyanine7 anti-human CD161 (Biolenged Cat# 339917), PE anti-human TCR Vα7.2 antibody (Biolenged Cat# 351705), FITC anti-human CD186 (CXCR6) antibody (Biolenged Cat# 356019). Data were analyzed using the FlowJo vX.07 software (Tree Star).

### Multiplex immunohistochemistry/immunofluorescence

Paraffin sections at baseline were obtained for immunostaining from the Department of Respiratory Disease, Thoracic Disease Center, The First Affiliated Hospital, Zhejiang University School of Medicine. Multiplex immunofluorescence staining was performed using an Opal Polaris 7 Color Multiplex IHC kit (NEL861001KT, Akoya Biosciences, Delaware, USA). The slides were dewaxed, and antigenic repair was performed using EDTA buffer (pH 9.0). The slides were washed with 3% H2O2 for 15 min, and sealed with 3% bovine serum albumin (BSA, B2064-100G, Sigma-Aldrich). The slides were incubated with specific primary antibodies targeting CD8 (Ab237709, Abcam; 1:2000), TCR Vα7.2(351726, Biolegend; 1:200), CXCR6 (NLS1102, Novus; 1:200) followed by Opal Polymer HRP Ms + Rb (Akoya Biosciences). Samples were subjected to optical fluorophore-conjugated tyramide signal amplification (Akoya Biosciences). The fluorescent dyes used for the detection of each antibody were Opal 570 (CXCR6) and Opal 520 (CD8). These steps were repeated until the cells were labeled with the expected markers and 4’,6-diamidino-2-phenylindole. Slices were visualized using the Vectra Polaris Quantitative Pathological Imaging System (Akoya Biosciences) and then analyzed using the InForm software (Akoya Biosciences).

### Statistical analysis

For scRNA-seq analysis, unpaired two-tailed Wilcoxon rank-sum tests were performed to assess the cell distribution differences between the two groups and unpaired two-tailed Student’s t-tests were employed to compare gene expression between the two groups. The Wilcox likelihood-ratio test was performed to identify differentially expressed genes, and genes expressed in more than 10% of the cells with more than 0.25 average log values were defined as differentially expressed genes.

Descriptive statistics were calculated to summarize the baseline characteristics and primary tumor response. Categorical variables were compared through the chi-squared test or Fisher’s exact test. Progression-free survival (PFS) and overall survival (OS) were estimated using Kaplan-Meier analysis and log-rank test. Hazard ratios (HRs) with corresponding 95% CIs were calculated utilizing a stratified Cox model. All statistical analyses were conducted using GraphPad Prism version 9 (GraphPad Software) and R software version 4.1.0 (R Project for Statistical Computing). Statistical significance was considered when *P* <0.05.

## Results

### The proportion of T cells is higher in responders than in non-responders

**Fig. 1:**
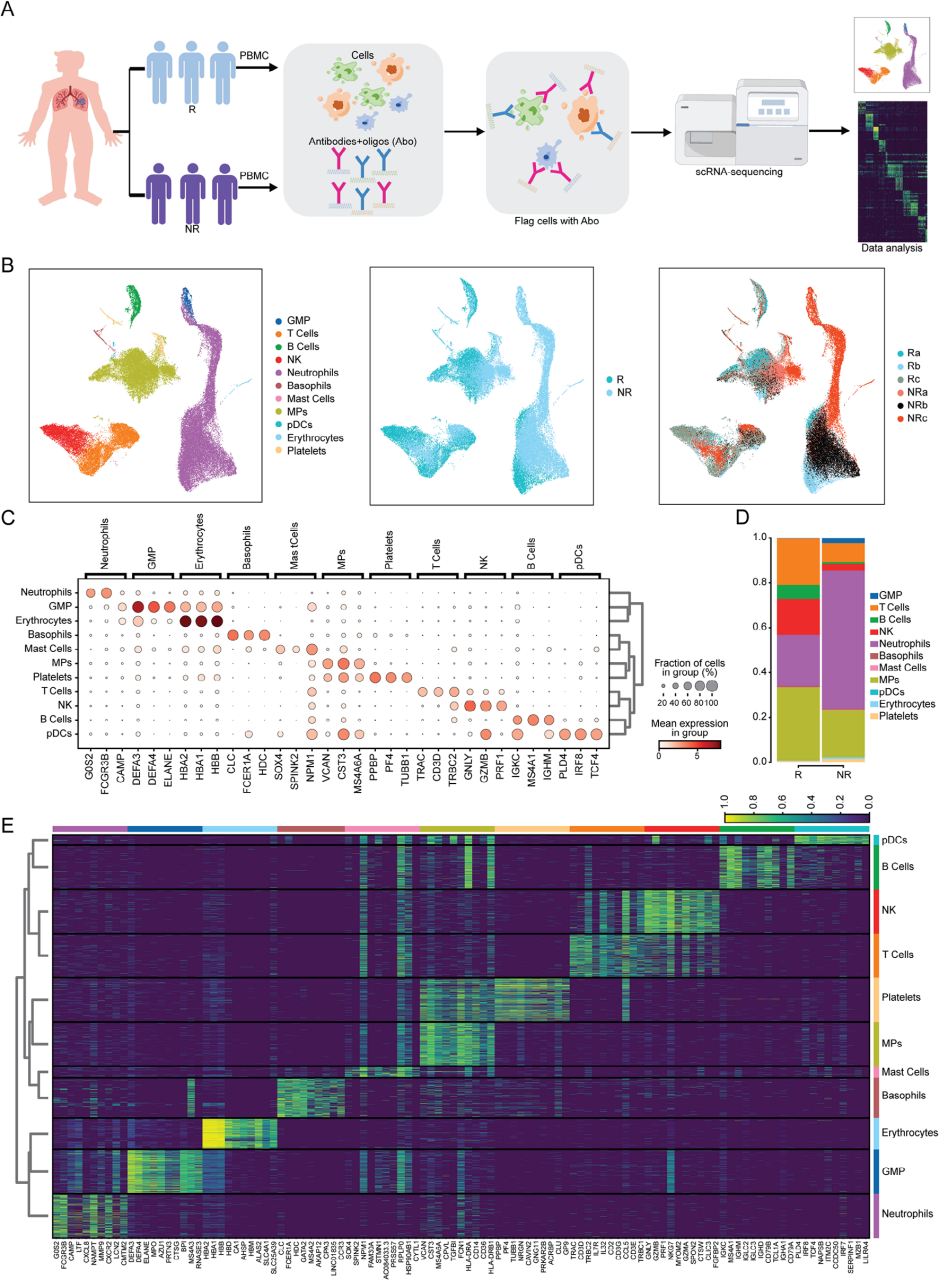
A higher proportion of T cells in responders. (**A**) The flowchart of scRNA-seq (peripheral blood samples of NSCLC patients were collected from 3 responders and 3 non-responders at baseline). (**B**) UMAP analysis plot showing cell clusters (left), classification according to anti-PD-1 therapy response (middle) and the derivation of samples (right). (**C**) The expression of canonical marker genes in different cell subtypes. (**D**) The proportion of cell subtypes in responders and non-responders. (E) Differentially expressed genes of different cell subtypes R: Responders; NR: Non-Responders. GMPs: Granulocyte-monocyte progenitor cells; NK: Natural killer cells; MPs: Mononuclear phagocytes; pDCs: Plasmacytoid dendritic cells

scRNA-seq was performed on the peripheral blood samples obtained from six patients with advanced NSCLC who received immunotherapy as first-line treatment, including three responders and three non-responders (Fig. 1A). The transcriptome of 56,805 cells (26,375 cells for responders and 30,430 cells for non-responders) was acquired. Based on the expression of canonical cell markers, peripheral blood cells were finally classified into 11 cell clusters by UMAP clustering analysis (Fig. 1B and C), including granulocyte-monocyte progenitor cells (GMPs), T cells, B cells, natural killer cells (NK), neutrophils, basophils, mast cells, mononuclear phagocytes (MPs), plasmacytoid dendritic cells (pDCs), erythrocytes and platelets. We compared the changes in cell proportions between responders and non-responders. The percentages of T Cells, B Cells, NK, and MPs were significantly increased in responders (Fig. 1D). Based on the scRNA-seq dataset, we analyzed the gene expression profile of each cell cluster, and the top ten differentially expressed genes were visualized using a heatmap. In addition to the well-recognized cell markers from previous studies, such as CD3 for T cells and MS4A1 for B cells [28], we found some other specifically/differentially expressed genes in different cell clusters. For example, the results showed that CD79B, and TCL1A were highly expressed in B cells, and SPON2 was highly expressed in NK cells (Fig. 1E). These genes may serve as new cell markers for corresponding immune cells. Since the importance of T Cells and NK in anti-PD-1/PD-L1 therapy has been reported previously, we conducted a comprehensive analysis of the cellular profiles and characterized these two cell cluster types in this study.

NK cells are crucial immune cells that maintain the stability of the immune environment by recognizing and attacking tumor cells and pathogen-infected cells. NK cells can also express PD-1, and immunotherapy against PD-1/PD-L1 may unleash the immune activity of NK cells [29–32]. There were 5170 NK cells in the samples, 4253 cells from responders, and 917 cells from non-responders. We compared differentially expressed genes, transcription factors, and cytokine expression profiles between responders- and non-responders-derived NK cells. NK cells in responders had a higher expression of CX3CR1 compared with non-responders (Supplementary Fig. 1A), which was reported to be involved in the recruitment of CD8+T cells, thus enhancing anti-PD-1/PD-L1 efficacy[33, 34]. Scenic analysis and cytokine expression profiling data showed that the transcription factors, ETS1 and ZNF394, and the cytokine, MIF, were highly expressed in responders-derived NK cells (Supplementary Fig. 1B-C). Masako et al. confirmed that the MIF-CD74 interaction can positively regulate the expression of PD-L1 in melanoma cells, thereby evading immune surveillance [35].

### CD8+MAIT cells are significantly increased in responders

**Fig. 2:**
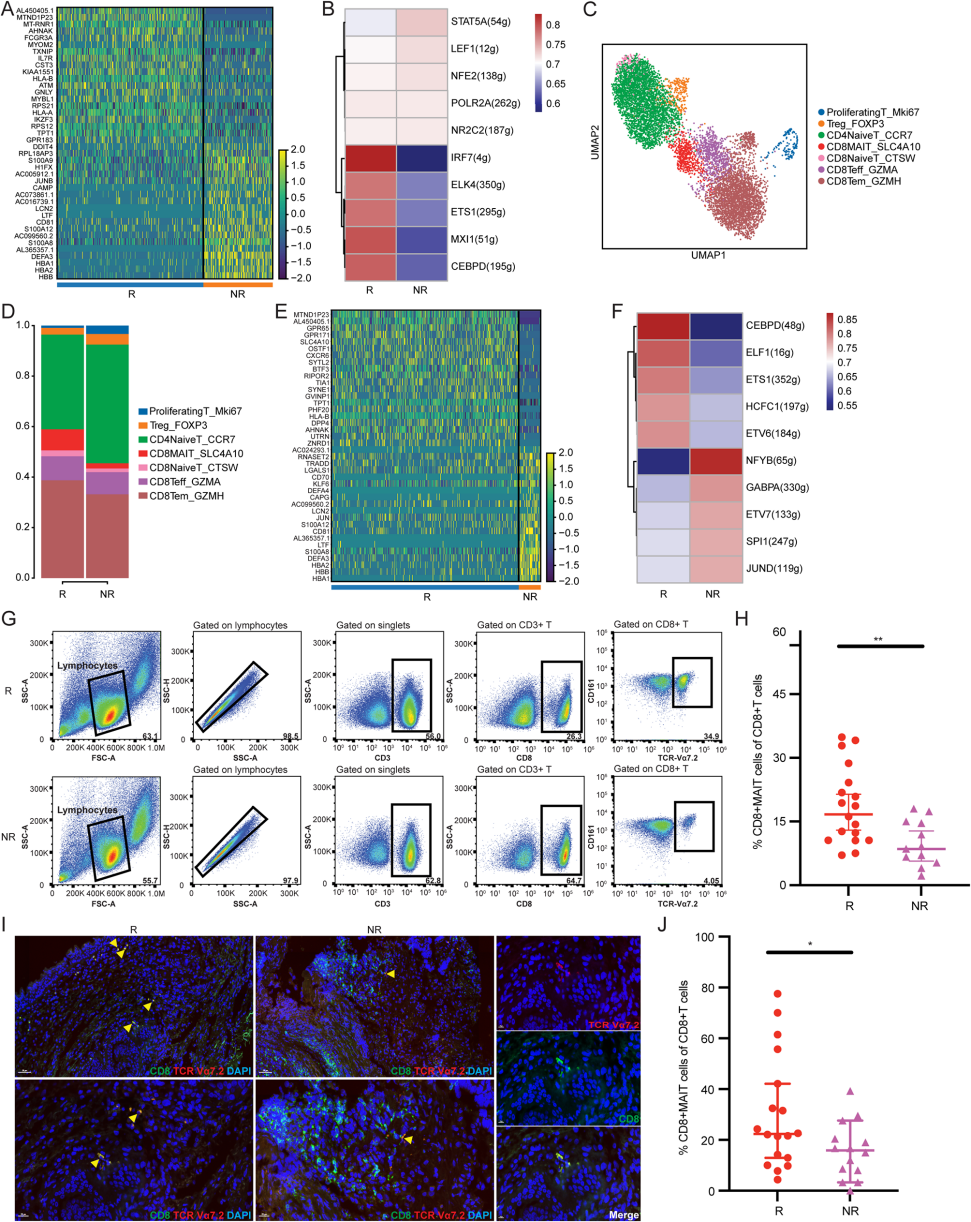
CD8+MAIT cells showed a significantly increased proportion in responders. (**A**) Differentially expressed genes between responders and non-responders-derived T cells. (**B**) Scenic analysis for responders and non-responders-derived T cells. (**C**) UMAP analysis plot showing T-cell subtypes. (**D**) The proportion of T-cell subtypes in responders and non-responders. (**E**) Differentially expressed genes between responders and non-responders in CD8+MAIT cells. (**F**) Scenic analysis for responders and non-responders-derived CD8+MAIT cells. (**G**) Flow cytometric analysis of responders and non-responders-derived PBMCs by labeling CD3, CD8, CD161, TCR α7.2(18 responders vs. 12 non-responders). (**H**) The percentage of CD8+MAIT cells in responders and non-responders-derived CD8+T cells from cytometric analysis (18.7% VS 10.0%, *P* = 0.0057). (**I**) Tumor tissues stained by immunofluorescence for CD8, TCR Vα7.2 in responders and non-responders. (**J**) Percentage of CD8+MAIT cells in CD8+T cells in responders and non-responders (30.1% VS 15.7%, *P* = 0.0347)

Tumor-associated T cells are triggered by their interaction with specific antigens on the surface of tumor cells directly or presented by APCs, thereby exerting their tumor identification and killing functions. This antigen-mediated cytotoxicity of T cells is the basis of tumor immunotherapy, including anti-PD-1/PD-L1 therapy [36, 37]. In this study, a differential gene expression analysis between responders- and non-responders-derived T cells was performed. AHNAK, FCGR3A, HLA-A, and HLA-B were upregulated in responders-derived T cells (Fig. 2A). These genes are associated with immune cell infiltration, PD-1/PD-L1 expression, and PD-1/PD-L1 blockade efficacy [38–43].  Next, scenic analysis was conducted on the T-cell clusters of both groups. IRF7 expression was significantly upregulated in responder-derived T cells (Fig. 2B). The expression of IRF7 was reported to be positively correlated with the enrichment of tumor-infiltrating CD8+T cells and M1 macrophages in glioma[44]; a similar result has been reported in colorectal cancer[45]. Together with our results, these findings suggest that patients with higher IRF7 expression may be more responsive to immunotherapy. Noman et al. showed that STAT1/IRF7 upregulates CCL5 and CXCL10 expression, leading to the enhanced efficacy of anti-PD-1/PD-L1 therapy[46]. Owing to the importance of T cells in immunotherapy, we explored the specific T cell type that was more likely to be associated with anti-PD-1/PD-L1 efficacy. Based on canonical marker genes and differentially expressed genes, T cells were further divided into seven subtypes: proliferating T, Treg, CD4+NaiveT, CD8+MAIT, CD8+NaiveT, CD8+Teff, and CD8+Tem (Fig. 2C). KEGG pathway analysis was conducted on the differentially expressed genes for each subtype, revealing gene-enriched signaling pathways. Several pathways possessed enriched genes in specific T-cell subtypes (Supplementary Fig. 2A-B). The proportion of CD8+MAIT cells was significantly higher in responders than in non-responders, indicating that CD8+MAIT cells may be associated with the efficacy of PD-1/PD-L1 blockade in patients with NSCLC (Fig. 2D). We analyzed the differentially expressed genes between CD8+MAIT cells from responders and non-responders. Consistent with the results from the T-cell clusters, GPR65, CXCR6, and DPP4 were also highly expressed in responders (Fig. 2E). The differentially expressed GPR65 [47], DPP4 [48], and CXCR6 [23, 24] are involved in the anti-cancer efficacy of PD-1 blockade by affecting the recruitment of tumor-associated macrophages or CD8 + T cells. Through scenic analysis, we identified the top five differentially expressed transcription factors in CD8+MAIT cells, among which CEBPD was significantly upregulated in responders (Fig. 2F). We collected more peripheral blood samples from responders and non-responders (18 and 12 samples, respectively) between September 2022 and June 2023, to determine the relationship between the characteristics of CD8+MAIT cells and sensitivity to anti-PD-1/PD-L1 immunotherapy in patients with NSCLC. Table 1 showed the clinical characteristics of the 30 patients. Then PBMCs were isolated for flow cytometry analysis. By labeling CD3, CD8, CD161, and TCR Vα7.2 in PBMCs, we sorted out the CD8+MAIT cell population (Fig. 2G). CD8+MAIT cells accounted for a significantly higher proportion of PBMCs-derived CD8+T cells in responders than in non-responders (18.7% VS 10.0%, *P* = 0.0057) (Fig. 2H). To explore the distribution of CD8+MAIT cells in the TME, we detected the infiltration of CD8+MAIT cells in tumor tissues from patients with NSCLC by multiplex immunofluorescence assay (18 responders and 14 non-responders) (Fig. 2I). Consistently, the percentage of CD8+MAIT cells in the tumor tissues of responders was significantly higher (30.1% VS 15.7%, *P* = 0.0347) (Fig. 2J).

**Fig. 3:**
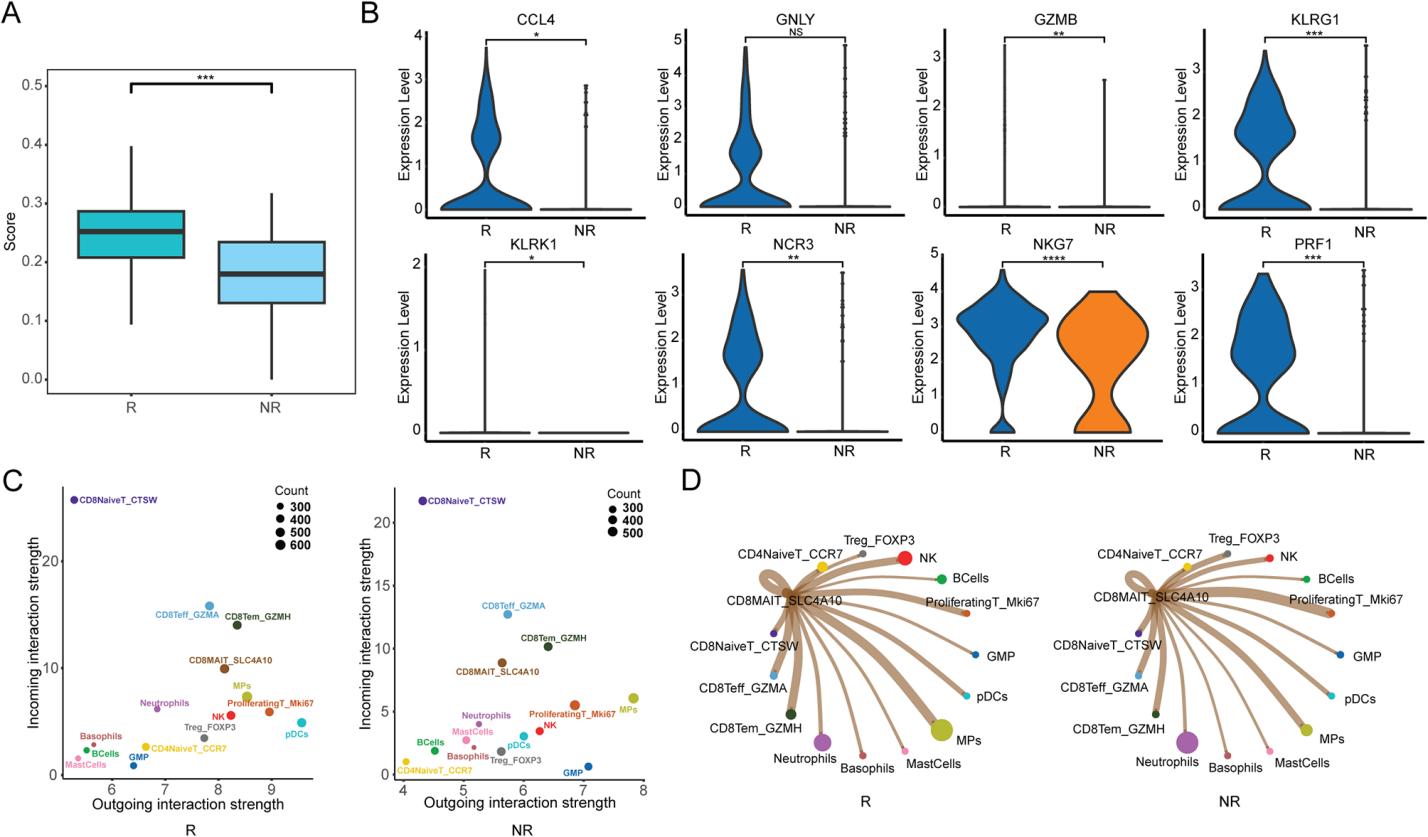
Responders in CD8+MAIT cells had higher expression of cytotoxicity-related genes. (**A**) Cytotoxicity scoring for responders and non-responders-derived CD8+MAIT cells. (**B**) Differentially expressed cytotoxic genes between responders and non-responders-derived CD8+MAIT cells. (**C**) The incoming and outgoing cell-cell interaction strength for responders and non-responders-derived cell subtypes. (**D**) The outgoing interaction pattern for responders and non-responders-derived CD8+MAIT.

**Table 1:** The characteristics of patients who received flow cytometry validation

	All patients (*n* = 30)	Responders (*n* = 18)	Non-responders (*n* = 12)	*P* value
**Gender**				>0.99
Male	28(93.3%)	17(94.4%)	11(91.7%)	
Female	2(6.7%)	1(5.6%)	1(8.3%)	
**Median age, years**	65(45–75)	65(45–74)	65(46–75)	0.54
**Disease stage**				0.46
IIIb-IIIc	12(40.0%)	6(33.3%)	6(50.0%)	
IV	18(60.0%)	12(66.7%)	6(50.0%)	
**Smoking status**				0.68
Never smoker	8(26.7%)	4(22.2%)	4(33.3%)	
Smoker	22(73.3%)	14(77.8%)	8(66.7%)	
**Histology**				0.33
Adenocarcinoma	14(46.7%)	11(61.1%)	4(33.3%)	
Squamous cell carcinoma	14(46.7%)	6(33.3%)	7(58.3%)	
Underdifferentiation NSCLC	2(6.7%)	1(5.6%)	1(8.3%)	
Immunotherapy regimens*				0.50
Pembrolizumab	15(50.0%)	9(50.0%)	6(50.0%)	
Sintilimab	5(16.7%)	4(22.2%)	1(8.3%)	
Atezolizumab	1(3.3%)	1(5.6%)	0(0.0%)	
Tislelizumab	8(26.7%)	4(22.2%)	4(33.3%)	
Toripalimab	1(3.3%)	0(0.0%)	1(8.3%)	
**PD-L1 expression**				0.94
≥ 50%	6(20.0%)	4(22.2%)	2(16.7%)	
1-49%	11(36.7%)	7(38.9%)	4(33.3%)	
<1%	4(13.3%)	2(11.1%)	2(16.7%)	
Untest	9(30.0%)	5(27.8%))	4(33.3%)	
**Sites of distant metastases**				0.08
Bone	7(23.3%)	3(16.7%)	4(33.3%)	
Lung	7(23.3%)	6(33.3%)	1(8.3%)	
Liver	1(3.3%)	0(0.0%)	1(8.3%)	
Adrenal	2(6.7%)	1(5.6%)	1(8.3%)	
CNS	4(13.3%)	4(22.2%	0(0.0%)	
Pleura	4(13.3%)	4(22.2%)	0(0.0%)	
mPFS	9.2 months	NR	4.0 months	<0.0001
mOS	NR	NR	NR	0.22

*All treatment stratigies were immunotherapy combined with chemotherapy. The final follow-up period was until November 30, 2023. NR: Not Reached.

### CD8+MAIT cells in responders show higher expression of cytotoxicity-related genes

Because of the significantly increased proportion of CD8+MAIT subtypes in responders and the important role of T cells in the tumor immune microenvironment, we focused on the molecular characteristics of CD8+MAIT cells in this study. We scored cytotoxicity by analyzing the expression of cytotoxicity-related genes in the R and NR groups. Compared to non-responders, responders had significantly higher expression of cytotoxicity-related genes in CD8+MAIT cells(Fig. 3A), including CCL4, KLRG1, PRF1, NCR3, NKG7, GZMB, and KLRK1(Fig. 3B). To study the interactions between different cells, we analyzed the incoming and outgoing signal strengths of each cell type in the responders and non-responders. CD8+MAIT had relatively strong cell-cell communication, and CD8+MAIT from responders had stronger outgoing signal strength than that from non-responders (Fig. 3C). Furthermore, CD8+MAIT from responders showed relatively strong signals for NK cells, MPs, and CD8+Tem cells (Fig. 3D). To further elucidate the specific ways in which different immune cell types interact, we analyzed the molecular pathways involved in cell-cell communication. The outgoing signals from responders-derived CD8+MAIT cells had relatively higher enrichment in CD99, ANNEXIN, CD48, SEMA4, LIGHT, CD6, and TGFβ-related molecular pathways (Supplementary Fig. 3).

### CD8+MAIT cells in responders cohort with higher CXCR6 expression

**Fig. 4:**
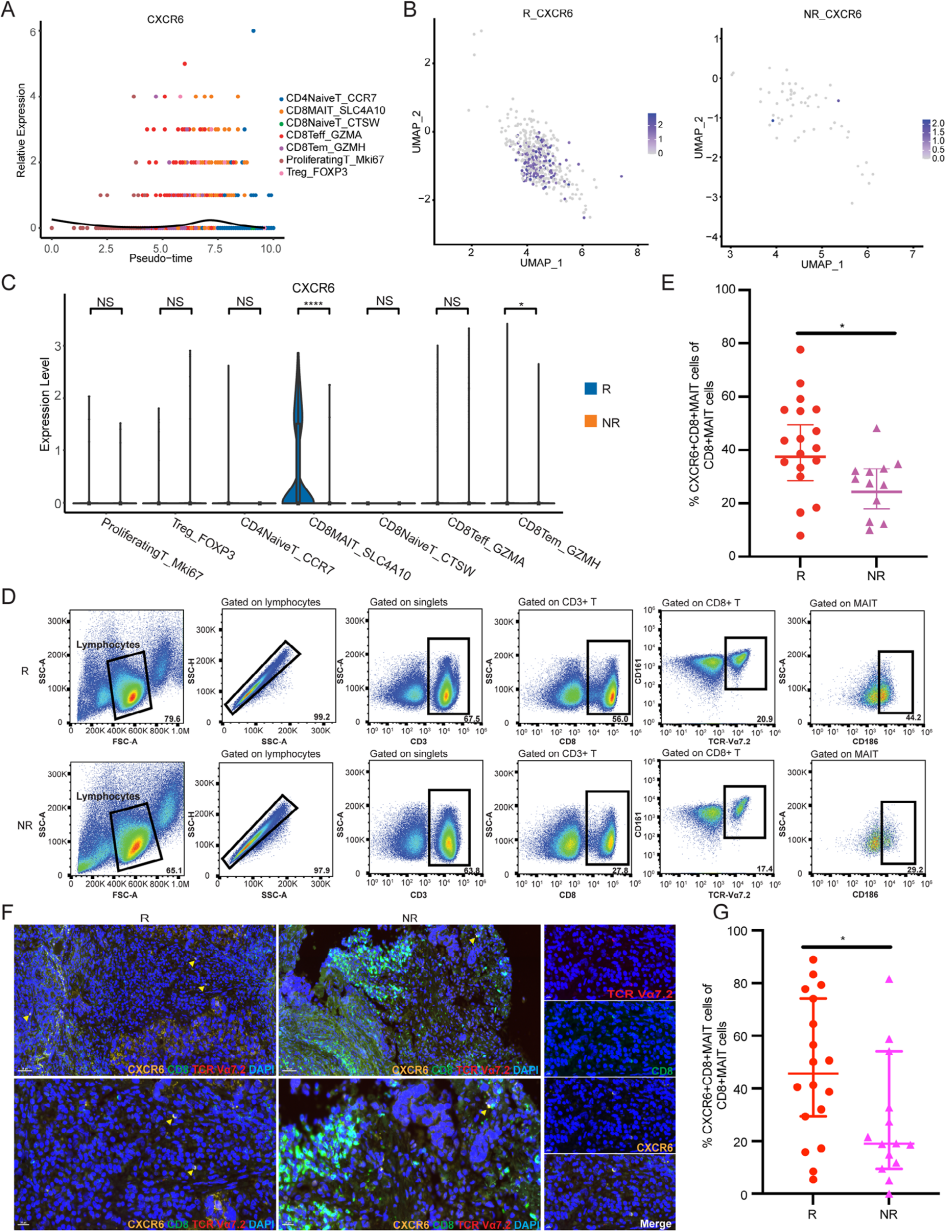
CD8+MAIT in responders cohort with higher CXCR6 expression. (**A**) Pseudo-time analysis for CXCR6 expression in different T-cell subtypes. (**B**) UMAP analysis for CXCR6 expression in responders and non-responders-derived CD8+MAIT cells. (**C**) Violin plots for CXCR6 expression in responders and non-responders-derived different T-cell subtypes. (**D**) Flow cytometric analysis of responders and non-responders-derived PBMCs by labeling CD3, CD8, CD161, TCR α7.2 and CD186 (18 responders vs. 12 non-responders). (**E**) The percentage of CD186+ (CXCR6+) CD8+MAIT cells in CD8+MAIT cells in responders and non-responders from cytometric analysis(*P* = 0.041). (**F-G**) Tumor tissues stained by immunofluorescence for CD8, TCR Vα7.2, CXCR6 in responders and non-responders and the summary data (47.44% VS 26.72%, *P* = 0.0274).

CXCR6 is a chemokine receptor that exerts its biological effects by binding to its ligand CXCL16[25]. CXCR6 enhances the response of colorectal cancer and melanoma cells to PD-1 blockade treatment[23]. Consistent with previous studies, CXCR6 was highly expressed in responders-derived CD8+MAIT cells (Fig. 2E). Therefore, we investigated the potential correlation between CXCR6 expression and the response of NSCLC cells to anti-PD-1/PD-L1 therapy. Using pseudo-time analysis, we confirmed that CXCR6 expression was relatively higher in the CD8+MAIT cluster (Fig. 4A). Moreover, through UMAP clustering analysis and violin plots, we found that CXCR6 was significantly upregulated in responder-derived CD8+MAIT cells, while it was barely expressed in non-responders (Fig. 4B-C). Flow cytometry revealed that the proportion of CD186+ (CXCR6+) CD8+MAIT cells was much higher in responders-derived CD8+MAIT cells compared to those from non-responders (42.1% VS 26.7%, *P* = 0.0119) (Fig. 4D-E). We conducted multiplex immunofluorescence staining of tumor tissues from both groups. An elevated percentage of tumor-infiltrating CXCR6+CD8+MAIT cells was observed (18 responders and 14 non-responders; 47.44% VS 26.72%, *P* = 0.0274) (Fig. 4F-G).

### CXCR6 in responders is associated with cytotoxicity of CD8+MAIT cells

**Fig. 5:**
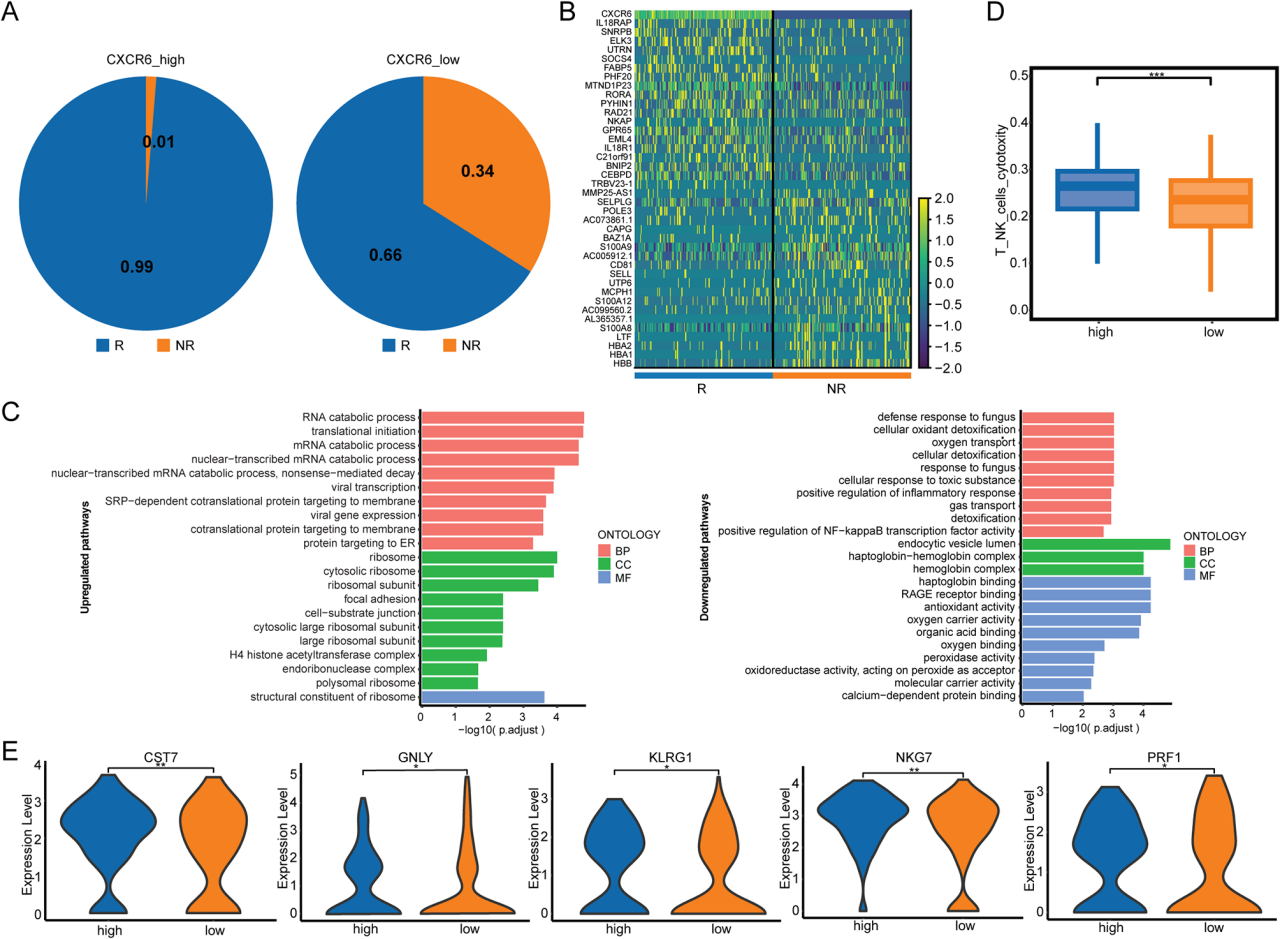
CXCR6 in responders was associated with cytotoxicity of CD8+MAIT cells. (**A**) The components of CXCR6-high and CXCR6-low CD8+MAIT cells. (**B**) Differentially expressed genes between CXCR6-high and CXCR6-low CD8+MAIT cells. (**C**) GO analysis for upregulated (left) and downregulated (right) pathways in CXCR6-high CD8+MAIT cells. (**D**) Cytotoxicity scoring for CXCR6-high and CXCR6-low CD8+MAIT cells. (**E**) Differentially expressed cytotoxic genes between CXCR6-high and CXCR6-low CD8+MAIT cells (*P *<0.05)

We found that 99% of the CXCR6 high-expressing CD8+MAIT cells were from responders (Fig. 5A). Based on the single-cell transcriptomic profile, we analyzed the differentially expressed genes between CXCR6-high and CXCR6-low CD8+MAIT cells, as shown in Fig. 5B. Additionally, we conducted GO pathway analysis to identify genes enriched in different signaling pathways in CD8+MAIT cells with different levels of CXCR6 expression. Differentially expressed genes were significantly enriched in RNA catabolic process, translational initiation, mRNA catabolic process, nuclear-transcribed mRNA catabolic process, and nonsense-mediated decay in CXCR6 high-expressing CD8+MAIT cells, while positive regulation of NF-kappa B translation factor activity-related genes were significantly enriched in CXCR6 low-expressing CD8+MAIT cells (Fig. 5C). Cytotoxicity scoring analysis confirmed that CXCR6 high-expressing CD8+MAIT cells exhibited significant enrichment of cytotoxicity-related genes compared to CXCR6 low-expressing CD8+MAIT cells (Fig. 5D), including CST7, GNLY, KLRG1, NKG7, and PRF1 (Fig. 5E).

### Levels of CD8+MAIT and CXCR6 +CD8+MAIT cells before anti-PD-1 therapy can identify responders

**Fig. 6:**
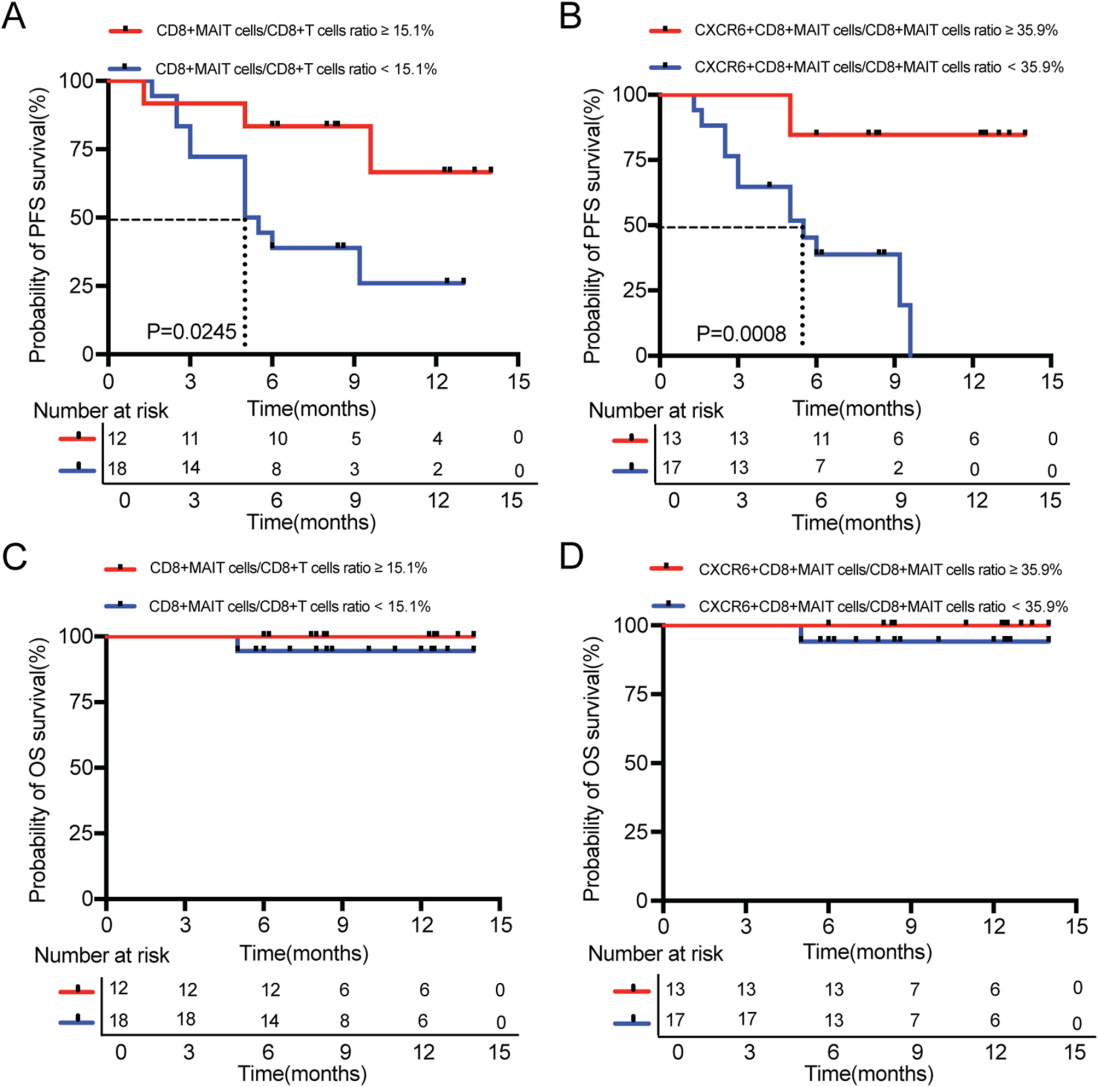
Levels of CD8+MAIT and CXCR6+CD8+MAIT cells before anti-PD-1 therapy identify responder patients. (**A**) CD8+MAIT cells/CD8+T cells ratio ≥15.1% had a better PFS than those patients with CD8+MAIT cells/CD8+T cells ratio <15.1% (NR vs. 5.3 months, *P* = 0.0245). (**B**) CXCR6+CD8+MAIT cells/CD8+MAIT cells ratio ≥35.9% had a better PFS than those patients with CXCR6 + CD8+MAIT cells/CD8+MAIT cells ratio <35.9% (NR vs. 5.5 months, *P* = 0.0008). (**C-D**) Kaplan–Meier survival analysis of the OS in patients with CD8+MAIT cells/CD8+T cells ratio ≥15.1% and < 15.1% (**C**), CXCR6 + CD8+MAIT cells/CD8+MAIT cells ratio ≥35.9% and < 35.9% (**D**)

Next, we investigated whether the levels of circulating CD8+MAIT and CXCR6+CD8+MAIT cells affected clinical prognosis as predictive biomarkers of the response to anti-PD-1 therapy in NSCLC. The final follow-up period was until November 30, 2023. Flow cytometry analysis revealed that within CD8+T cells, the median CD8+MAIT cells level in the population of patients with metastatic NSCLC was 15.1%; thus, this value was used as a cutoff to stratify patients. The median level of CXCR6+CD8+MAIT cells in CD8+MAIT cells of patients with metastatic NSCLC was 35.9%. Figure 6A showed that patients with a CD8+MAIT cells to CD8+T cells ratio ≥15.1% had a better PFS than those with a ratio  <15.1% (NR vs. 5.3 months, *P* = 0.0245, Log-rank Mantel-Cox test). Figure 6B showed that patients with a CXCR6+CD8+MAIT cells to CD8+MAIT cells ratio ≥ 35.9% had improved PFS than those with a ratio < 35.9% (NR vs. 5.5 months, *P* = 0.0008, Log-rank Mantel-Cox test). Kaplan-Meier survival analysis did not show significant changes in OS of patients with CD8+MAIT cells to CD8+T cells ratio ≥15.1% and < 15.1%, CXCR6+CD8+MAIT cells to CD8+MAIT cells ratio ≥35.9% and < 35.9% because of the short survival follow-up time (Fig. 6C-D).

### The role of CD8+MAIT cells in NSCLC immunotherapy may be achieved through the CXCR6-CXCL16 axis

**Fig. 7:**
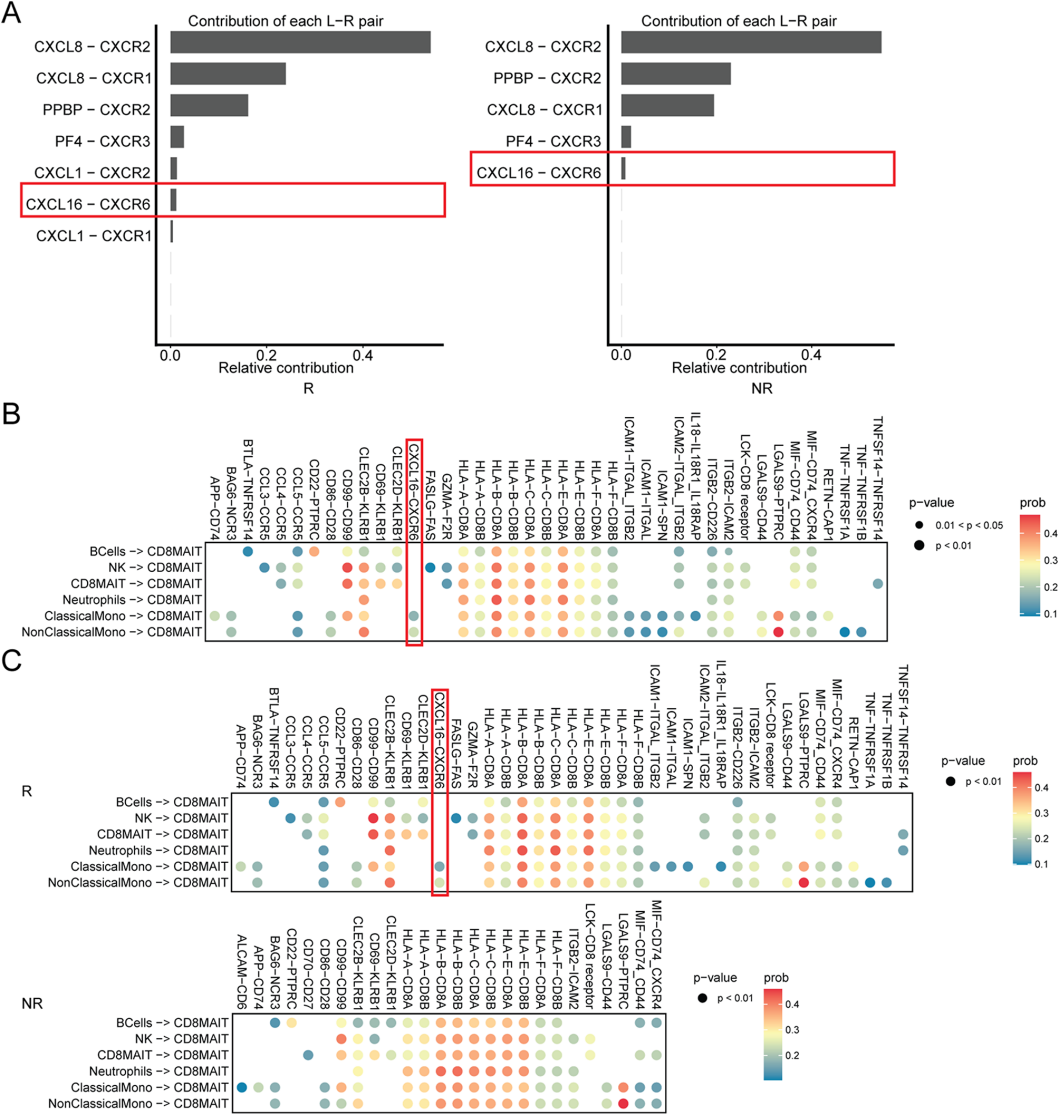
The role of CD8+MAIT cells in lung cancer immunotherapy may be achieved by CXCR6-CXCL16 axis. (**A**) The contributions of main ligand-receptor pairs in cell-cell interaction of responders and non-responders-derived CD8+MAIT cells. (**B**) Ligand-receptor pairs participated in cell-cell interaction of CD8+MAIT cells. (**C**) Ligand-receptor pairs participated in cell-cell interaction of responders and non-responders-derived CD8+MAIT cells

CXCL16 is a unique CXCR6 ligand. However, the role of CXCL16 in anti-PD-1/PD-L1 therapy for lung cancer remains unknown. Given the importance of CXCR6 in lung cancer immunotherapy, we investigated whether the function of CXCR6 is mediated by the CXCL16-CXCR6 axis. In this study, we analyzed the ligand-receptor pairs that participate in cell-cell communication in CD8+MAIT cells based on scRNA-seq datasets. Relative contribution of CXCL16-CXCR6 to cell-cell communication was higher in responder-derived CD8+MAIT cells (Fig. 7A). Next, to identify the potential cell types in the circulation that could interact with CD8+MAIT cells via CXCL16-CXCR6 pairs, we analyzed the scRNA-seq transcriptomic profiles by cell-cell interaction analysis. Only classical/non-classical monocytes had a significant predictive value for interaction with CD8+MAIT cells by producing CXCL16 as a ligand combined with CXCR6 on CD8+MAIT (Fig. 7B; Supplementary Fig. 4A). Cell-cell interaction analysis was performed separately in responders and non-responders-derived CD8+MAIT cells. Classical/non-classical monocytes could interact with responders-derived CD8+MAIT cells via CXCL16-CXCR6, while non-responders-derived CD8+MAIT cells did not (Fig. 7C; Supplementary Fig. 4B-C).

## Discussion

Anti-PD-1/PD-L1 therapy has dramatically changed the landscape of cancer treatment. Significant and durable drug responses in different types of cancers have been documented. However, more than half of these patients do not benefit from immunotherapy. Therefore, screening of defined predictive biomarkers for therapeutic efficacy is a crucial challenge that needs to be addressed. Although existing researches have suggested correlations between PD-L1 expression or PD-1/PD-L1 interaction, tumor mutation burden, and tumor-infiltrating lymphocytes with anti-PD-1/PD-L1 therapy efficacy [49, 50], no biomarker has been identified that is sufficient to stratify patients who would benefit from immunotherapy effectively. In this study, we mapped the peripheral blood-derived PBMCs transcriptional landscape of anti-PD-1/PD-L1 responsive and non-responsive patients with NSCLC by scRNA-seq analysis.

MAIT cells, important for the modulation of infection, inflammation, autoimmunity, and cancer, are enriched in both mucosal and non-mucosal tissues in humans. Since the discovery of MAIT cells, abundant researches have focused on their correlation with the TME. However, the role of MAIT cells in solid tumors remains unclear. In this study, using RNA-seq and clustering analysis of peripheral blood samples from patients with NSCLC before treatment, we found significant enrichment of CD8+MAIT cells in patients who responded to anti-PD-1 therapy. Previous studies have shown that the percentage of CD8+MAIT cells was higher in the peripheral blood of patients with lung cancer, and the abundance of CD38+CD8+MAIT cells was negatively correlated with the PFS of patients with lung cancer [51]. However, Lao et al. found that several T-cell subsets, including MAIT cells, were enriched in the responder-derived peripheral blood of patients with NSCLC after immunotherapy but no difference was observed in non-responders [52]. Additionally, a recent study by Shi et al. found that MAIT cells could migrate from peripheral blood to tumor tissue through the CCR6-CCL20 axis and that tumor-infiltrating MAIT-17s and MAIT-IFNGR phenotypes were associated with the sensitivity of patients with NSCLC to anti-PD-1 immunotherapy [53]. Combined with the results of our study, we speculate that the percentage of CD8+MAIT cells in the peripheral blood of patients with NSCLC may be related to the sensitivity to immunotherapy and may serve as a predictive biomarker for immunotherapy efficacy in patients with lung cancer. Furthermore, we also verified the elevated proportion of CD8+MAIT cells by flow cytometric analysis in PBMCs and multiplex immunofluorescence in tumor tissues. Through cytotoxicity scoring analysis, we found that several cytotoxicity-related genes were highly expressed in CD8+MAIT cells from responders, including CCL4, GNLY, KLRG1, PRF1, NCR3, NKG7, GZMB, and KLRK1. We observed longer PFS in CD8+MAIT-enriched patients. Taken together, CD8+MAIT cells are enriched in responders-derived peripheral blood, and these cytotoxic CD8+MAIT cells may increase the sensitivity of patients with NSCLC to anti-PD-1 therapy by promoting the expression of corresponding cytotoxic molecules, thereby leading to a better prognosis. In this study, we explored the potential mechanisms by which CD8+MAIT cells affected efficacy of immunotherapy in patients with NSCLC.

CXCR6 is a potential prognostic biomarker in lung adenocarcinoma [54]. Karaki et al. elucidated that the loss of CXCR6 could lead to a defect in CD8+T cell recruitment in the lungs [24]. The lack of cytotoxic CD8+T cells in the TME is one of the main reasons for resistance to tumor immunotherapy. Further studies are required to explore the relationship between CXCR6 expression and immunotherapy. In this study, we found that CXCR6 was enriched in CD8+MAIT cells, and CXCR6 was significantly upregulated in responders-derived CD8+MAIT cells. The results were further confirmed by flow cytometric analysis and multiplex immunofluorescence assay in PBMCs and tumor tissues, respectively. Hence, CXCR6 may be associated with sensitivity to immunotherapy in NSCLC. In a previous study, Wang et al. confirmed that CXCR6 was specifically highly expressed in CD8 + T cells and correlated positively with the prognosis of patients with colorectal cancer, while the deficiency of CXCR6 led to lower anti-PD-1 efficacy [23]. We found that responder-derived CD8+MAIT cells with higher CXCR6 expression had significantly higher cytotoxicity scores and increased CST7, GNLY, KLRG1, NKG7, and PRF1 expression. Considering the results of this and previous studies, we confirmed that CXCR6 can mediate the cytotoxicity of CD8+MAIT cells to malignant cells in NSCLC immunotherapy, thereby increasing sensitivity to immunotherapy. Survival analysis supported this hypothesis, indicating that patients with NSCLC with a higher percentage of CXCR6+CD8+MAIT cells had significantly prolonged PFS.

CXCL16 is a special ligand of CXCR6. CXCL16 is overexpressed in patients with lung cancer, while its expression is predominantly decreased after chemotherapy and VEGF-targeted therapy, which is associated with better response rates [55]. However, the role of CXCL16 in lung cancer immunotherapy remains unclear. Herein, cell-cell interaction analysis revealed that classical/non-classical monocytes could interact with CD8+MAIT cells in responders through the CXCL16-CXCR6 axis but not in non-responders. Our results suggest that the CXCR6-CXCL16 axis mediates the enhanced immunotherapeutic efficacy of CD8+MAIT cells in NSCLC. However, further exploration is needed in the future.

This study has some limitations that warrant consideration. For instance, although we found that the features of CD8+MAIT cells from patients prior to treatment may be associated with immunotherapy efficacy, the characteristics of CD8+MAIT cells during treatment were not explored. The results of this study were based only on the analysis of clinical samples, such as peripheral blood and tumor tissues, and have not been thoroughly studied and verified by functional experiments in cells and in vivo using animal models. In addition, the follow-up time and sample size of the validation cohort is limited, more data need to be collected afterwards for further verification. With the development of new technologies for MAIT cell isolation and expansion and the construction of chimeric animal models, it provides us an opportunity to investigate the specific molecular mechanisms by which CD8+MAIT cells affects the efficacy of immunotherapy in patients with NSCLC.

## Conclusion

In summary, we demonstrated that the CD8+MAIT subset is specifically enriched in the peripheral blood of immunotherapy-responsive patients with NSCLC. The correlation between CD8+MAIT cells expression and immunotherapy sensitivity may be ascribed to high CXCR6 expression. Here, for the first time, we elucidated that CD8+MAIT cells in the peripheral blood could be a potential predictive biomarker for immunotherapy efficacy.

### Electronic supplementary material

Below is the link to the electronic supplementary material.

Supplementary Material 1

## Data Availability

The data that support the findings of this study are available from the corresponding author (zhoujy@zju.edu.cn) upon reasonable request. Additional data are available as supplementary material.
