# Supplementary material for: CXCR6-positive circulating mucosal-associated invariant T cells can identify patients with non-small cell lung cancer responding to anti-PD-1 immunotherapy
Source: J Exp Clin Cancer Res. 2024 May 3;43:134. doi: 10.1186/s13046-024-03046-3 (PMC11067263; doi:10.1186/s13046-024-03046-3)
Supplement: Supplementary file 1 — Supplementary Material 1 [file 13046_2024_3046_MOESM1_ESM.docx]

**Supplementary data**

**CXCR6-positive circulating mucosal-associated invariant T cells can identify patients with non-small cell lung cancer responding to anti-PD-1 immunotherapy**

Jingjing Qu^1,2#^, Binggen Wu^1,2#^, Lijun Chen ^3^, Zuoshi Wen^3^, Liangjie Fang^1,2^, Jing Zheng^1,2^, Qian Shen^1,2^, Jianfu Heng^5^**^*^**, Jianya Zhou^1,2^**^*^**, Jianying Zhou^1,2^

1. Department of Respiratory Disease, Thoracic Disease Center, The First Affiliated Hospital, Zhejiang University School of Medicine, Hangzhou, Zhejiang 310003, P. R. China

2. The Clinical Research Center for Respiratory Diseases of Zhejiang Province, Hangzhou, Zhejiang 310003, P. R. China

3. State Key Laboratory for Diagnosis and Treatment of Infectious Diseases, National Clinical Research Center for Infectious Diseases, The First Affiliated Hospital, Zhejiang University School of Medicine, Hangzhou, Zhejiang 310003, P.R. China

4. Department of Cardiology, The First Affiliated Hospital, The First Affiliated Hospital, Zhejiang University School of Medicine, Hangzhou, Zhejiang 310003, P. R. China

5. Department of Clinical Pharmaceutical Research Institution, Hunan Cancer Hospital/the Affiliated Cancer Hospital of Xiangya School of Medicine, Central South University, Changsha, Hunan, 410013, P. R. China

# These authors contributed equally

* Correspondence should be addressed to **Prof. Jianya Zhou**, Department of Respiratory Disease, The First Affiliated Hospital, Zhejiang University School of Medicine, Hangzhou, Zhejiang 310003, P.R. China. [zhoujy@zju.edu.cn](mailto:zhoujy@zju.edu.cn) and **Prof. Jianfu Heng**, Department of Clinical Pharmaceutical Research Institution, Hunan Cancer Hospital/the Affiliated Cancer Hospital of Xiangya School of Medicine, Central South University, Changsha, Hunan, 410013, China, hengjianfu106@163.com.

**Supplementary Figure**

**
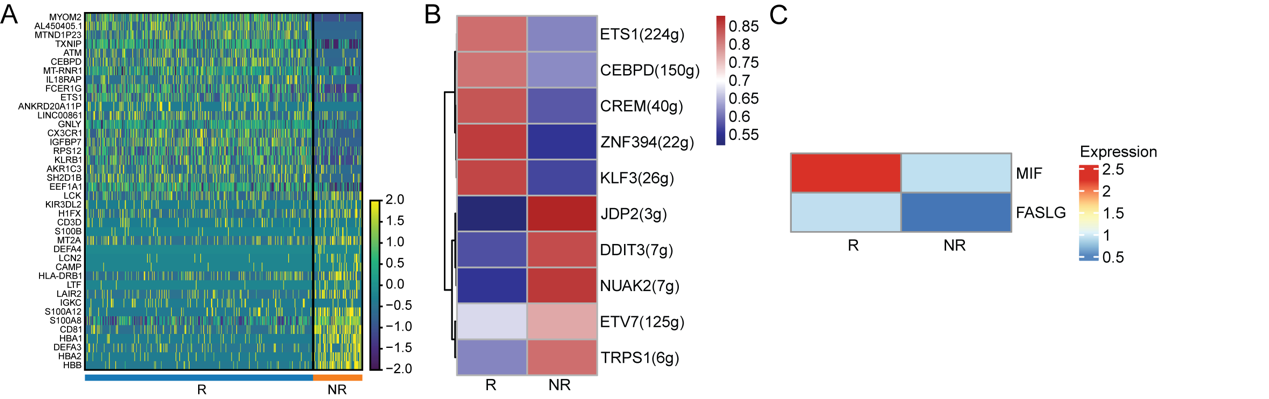
**

**Supplementary Figure 1: Higher proportion of NK cells in responders.** (A) Differentially expressed genes between responder and non-responders-deried NK cells. (B) Scenic analysis for responder/non-responders-deried NK cells. (C) Differentially expressed cytokines between responder and non-responders-deried NK cells.


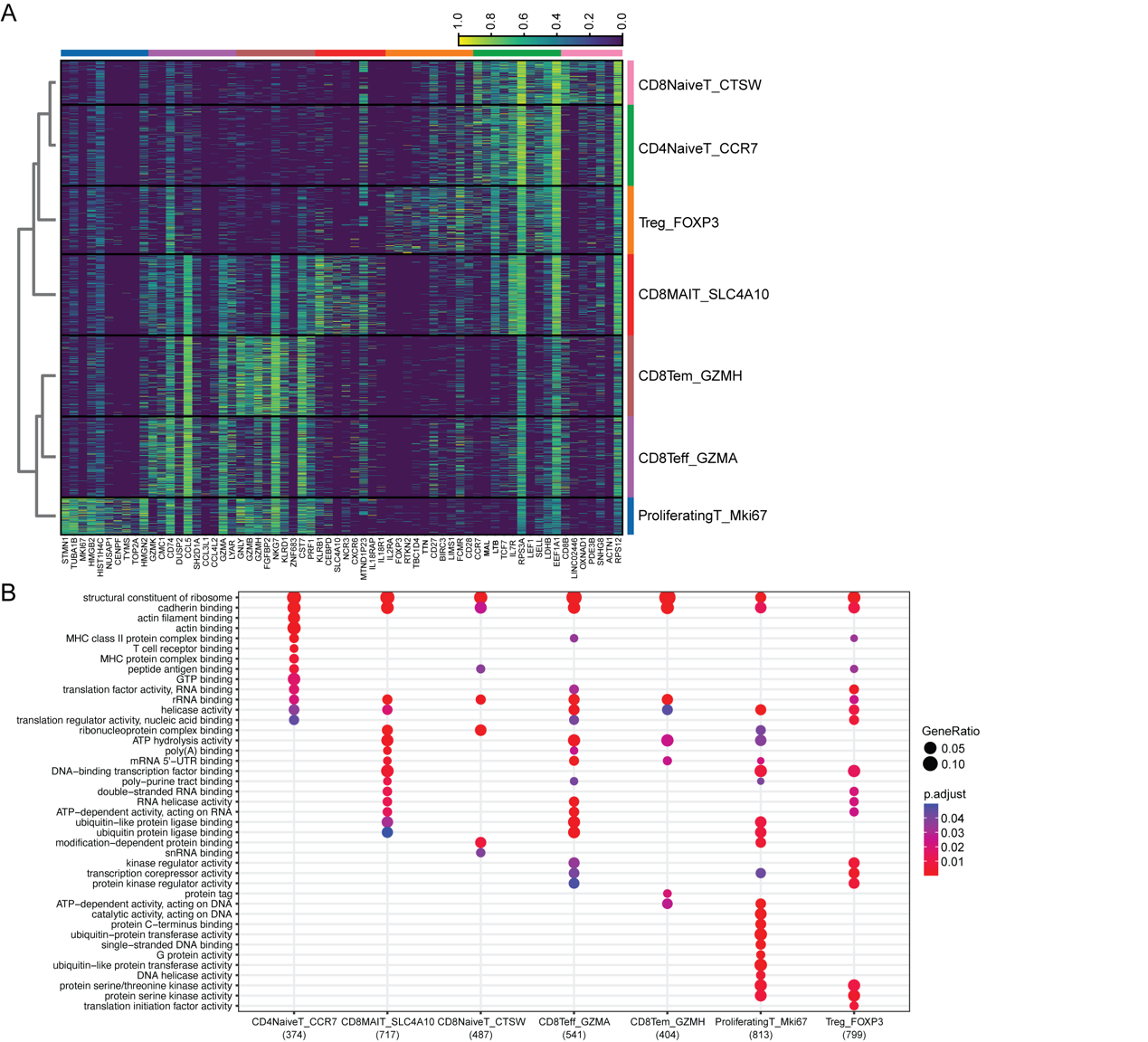


**Supplementary Figure 2: The DEG enriched pathways in different T cell subtypes.** (A) Differentially expressed genes of different T cell subtypes (B) KEGG analysis for different T cell subtypes.


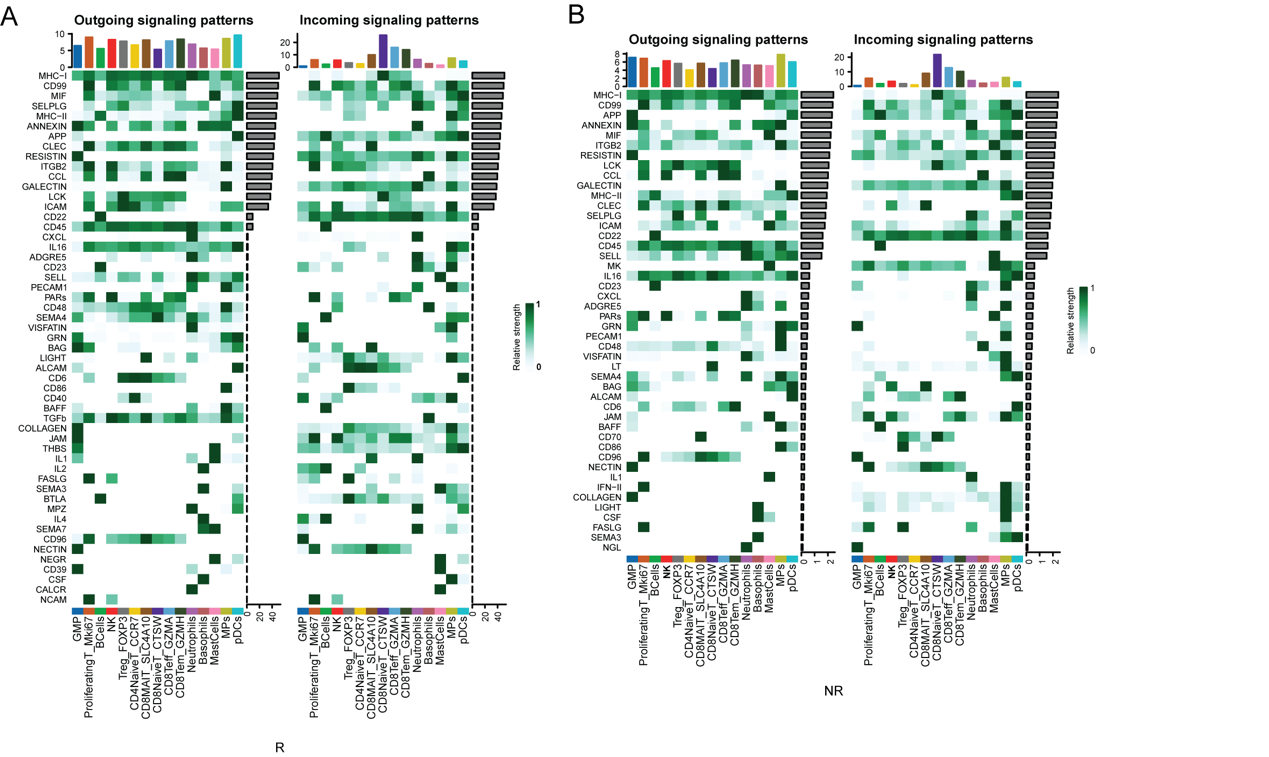


**Supplementary Figure 3: Cell-cell communication analysis.** (A-B)The incoming and outgoing cell-cell interaction-related pathways for responder/non-responders-deried different cell subtypes(columns on the right of the heatmap represent the total signal strength of each signaling pathway; columns on the top represent the total signal strength related to each cell type).


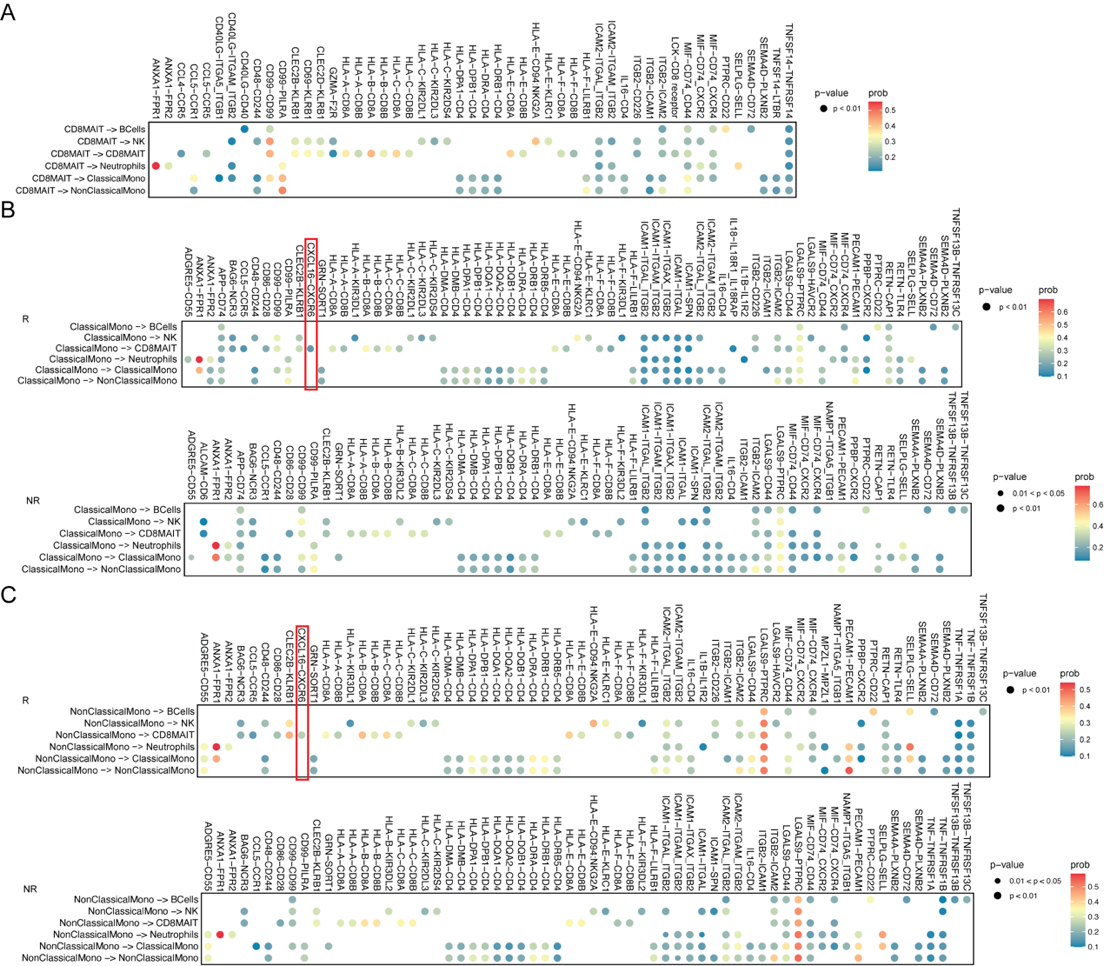


**Supplementary Figure 4: CellChat analysis in CD8MAIT and classical/non-classical monocytes.** (A) Ligand-receptor pairs participated in cell-cell interaction of CD8MAIT cells (CD8MAIT as sender). (B-C) Ligand-receptor pairs participated in cell-cell interaction of classical/non-classical monocytes in responders and non-responders(classical/non-classical monocytes as senders).
